# Controlling the Oxygen Defects Concentration in a Pure BiFeO_3_ Bulk Ceramic

**DOI:** 10.3390/ma15196509

**Published:** 2022-09-20

**Authors:** Anton Tuluk, Hans Brouwer, Sybrand van der Zwaag

**Affiliations:** 1 Novel Aerospace Materials (NovAM) Group, Faculty of Aerospace Engineering, Delft University of Technology, Kluyverweg 1, 2629 Delft, The Netherlands; 2 Materials Science and Engineering Department, Faculty of Mechanical, Maritime and Materials Engineering, Delft University of Technology, Mekelweg 2, 2628 Delft, The Netherlands

**Keywords:** bismuth ferrite, electrical conductivity, impedance spectroscopy, oxygen defects, atmosphere

## Abstract

BiFeO_3_ is a multiferroic material with a perovskite structure that has a lot of potential for use in sensors and transducers. However, obtaining pure single-phase BiFeO_3_ ceramic with a low electrical conductivity via solid-state reactions remains a problem that limits its application. In this work, the suppression of secondary phases in BiFeO_3_ was studied by varying the compositional parameters and the sintering temperature. The addition of 1% Bi_2_O_3_ to the stoichiometric precursor mixture prevented the formation of secondary phases observed when sintering stoichiometric precursors. The pure phase ceramic had a p-type conductivity and a three-decade lower electrical conductivity as measured by impedance spectroscopy. Annealing of optimally synthesized material at different partial pressures of oxygen in an oxygen–nitrogen gas atmosphere showed that the reason for this type of conductivity lies in the high concentration of defects associated with oxygen. By annealing in various mixtures of nitrogen and oxygen, it is possible to control the concentration of these defects and hence the conductivity, which can go down another two decades. At a pO_2_ ≤10%, the conductivity is determined by intrinsic charge carriers in the material itself.

## 1. Introduction

Bismuth ferrite (BiFeO_3_), and the solid solutions based on it, are promising multiferroic materials for use in sensors and transducers due to its high Curie temperature, T_C_, of 825 °C [1]. In principle, this allows for the possibility of using it as a lead-free high-temperature piezoceramic [2]. Although bismuth ferrite was discovered already in the late 1950s, obtaining it in a single phase form remains a challenge due to the easy formation of the stable secondary phases of Sillenite (Bi_25_FeO_39_) and Mullite (Bi_2_Fe_4_O_9_) [3]. The presence of these secondary phases leads to large electrical leakage currents, which imposes serious restrictions on its use in actual sensor applications. Due to the high coercive field required to pole this material, it is important to increase its insulating properties [4]. Therefore, before studying the electrical properties, it is necessary to obtain high-density ceramics as free from secondary phases as possible. In previous works, various methods of obtaining pure bismuth ferrite were used, such as solid-state synthesis [5,6], rapid liquid reaction [7], mechano-chemical activation [8], and wet-chemical methods [9], as well as others. However, the formation of secondary phases could not be avoided in any of them. To eliminate secondary phases present after synthesis, leaching in nitric acid [10] was proposed, which shows good results, but probably leads to uncontrolled changes in the composition of the material, which will impact the defect chemistry. Given the versatility and cost-effectiveness in the synthesis of inorganic solid materials [11] and the recent developments of the method [12,13,14,15], in this work, solid-state sintering at different temperatures and using non-stoichiometric powder mixtures are employed to try and synthesize pure-phase BiFeO_3_ ceramic samples.

For pure and impure materials alike, the defect configuration of bismuth ferrite is very dependent on sample preparation [10], which of course, affects the conductivity of the material. However, the nature of defects is mainly studied theoretically [16,17,18]. As shown in previous works, the high-temperature sintering of Bi-based systems can lead to the creation of oxygen vacancies by the release of electrons [19]. The increase in oxygen vacancies can also induce a change in the valence state of Fe^3+^. If the released electrons bind with the Fe^3+^ ions in the system, a charge transformation from Fe^3+^ to Fe^2+^ will take place.

The oxygen defect nature and level are probably not only affected by the obvious sintering conditions but also by the annealing environment, in particular, the oxygen partial pressure, during high-temperature processing. However, no systematic studies of the oxygen partial pressure, pO_2_, during high-temperature processing on the electrical conductivity can be found in the literature; this may be due to the notorious difficulty of obtaining pure-phase bulk BiFeO_3_ ceramics with low leakage currents and thermal instability of this phase at a higher temperature, which makes it difficult to reproduce the results due to changes in the sample during the annealing process. Furthermore, annealing samples at high temperatures under well-controlled oxygen levels is non-trivial and requires special equipment usually not available in most laboratories.

Electrical impedance spectroscopy is a useful approach to studying the electrical properties of electroceramics, including ferroelectrics [20]. The impedance data can be used to calculate the DC conductivity and to distinguish between long-range polarization and charge carrier diffusion processes. Furthermore, the impedance data can be presented in the form of an electrical modulus, which gives more information about the short-range polarization processes in the material. The electrical modulus can give insight into the dielectric processes occurring inside the material [21]. The low-frequency side of the peak in electrical modulus represents the range of frequencies in which the charge carriers can move over a long distance (charge carriers can perform successful hopping from one site to the neighboring site). The high-frequency side of the electric modulus represents the range of frequencies in which the charge carriers are spatially confined to their potential wells, and thus can only make localized motions inside the well. The frequency at which the peak occurs is an indication of the transition from long-range to short-range conduction. Asymmetrical behavior of the electrical modulus peak demonstrates non-Debye-type relaxation phenomena, which are in good agreement with the observations from the conductivity spectrum. An increase in the frequency of the peak is consistent with an increasing activation energy of conductivity representing long-range movement. Although the impedance spectra or electrical modulus do not uniquely specify the nature of the observed polarization and conductivity phenomena, it is possible to obtain the correlation between certain types of defects and conductivity or polarization by comparing differently processed or annealed samples and inferring the mechanism.

The present work is devoted to the optimization of making pure BiFeO_3_ ceramics by classic solid-state reaction for subsequent investigations of the defect chemistry. The effect of the calcination temperature on phase formation is studied. Furthermore, the influence of excess Bi_2_O_3_ addition is investigated. Impedance spectroscopy on the as-made ceramics is performed to investigate the influence of phase purity on the electrical properties. Furthermore, to vary and control the concentration of oxygen-related defects, optimally sintered samples were annealed in nitrogen–oxygen gas mixtures with different oxygen concentrations during heating, holding at 750 °C, and subsequent cooling to room temperature. The oxygen concentration was maintained throughout the entire experiment.

## 2. Experimental Procedures

BiFeO_3_ samples with and without 1 mol% excess Bi_2_O_3_ were prepared by a solid-state reaction from pre-milled Bi_2_O_3_ and Fe_2_O_3_ powder in the appropriate ratios. Milling was performed using 2 mm Y_2_O_3_-stabilized ZrO_2_ balls in hexane using a Retsch PM100 planetary ball mill. The powders were dried and calcined at a range of temperatures between 700 and 825 °C in air for 1 h with a heating rate of 600 °C/h. The reacted powder with an average particle size of 1 μm was ground again, and cold pressed into pellets using a uniaxial press. After that, the samples were sintered at temperatures between 750 °C to 850 °C in air for 1 h at a heating rate of 600 °C/h. (see Figure S1 of the Supplementary Information for an photograph of a typical sample). The block diagram of the sintering experiments is shown in Figure S2 of the Supplementary Information section.

Optimally sintered samples (1% excess Bi_2_O_3_ and sintered at 750 °C) were then annealed in a quartz tube furnace at 750 °C for 1 h in a mixture of pure nitrogen and oxygen gases with different oxygen fractions (100%, 20%, 10%, 1%). The flows were controlled by Bronkhorst Nederland mass flow controllers of type F-201CV-20-RAD-22-V. The N_2_ and O_2_ were supplied by Linde, the Netherlands, and were of quality 5 N. Nitrogen was filtered for hydrocarbons, water, and oxygen. Oxygen was filtered for hydrocarbons and water. The filters were supplied by Messer Griesheim, Germany. The flows for the different annealing are shown in Table 1.

In order to achieve 1% oxygen concentration in the gaseous atmosphere in the quartz tube furnace, the nitrogen gas flow had to be increased to 2970 mL/min because the lowest controlled setting for the oxygen mass flow controllers was 20 mL/min. The other experiments were carried out at a lower total gas flow in order to reduce gas consumption. The gas conditions were maintained during heating, holding, and cooling. The block diagram for the annealing environment experiments is given in Figure S3 in the Supplementary Information section.

The phase purity of the crushed BiFeO_3_ samples was analyzed by X-ray diffraction analysis using a Rigaku Miniflex 600 tabletop diffractometer and Cu Kα radiation at room temperature. The density was determined by Archimedes’ method in water. The microstructures of sintered ceramics were investigated using scanning electron microscopy (SEM), using a Jeol JSM-7500F field emission scanning electron microscope. For electrical measurements, gold electrodes were deposited on the ceramics by DC sputtering using a Quorum Q300T sputter coater. Electrical measurements were performed with a Novocontrol Alpha Dielectric Analyser in the frequency range from 1 Hz to 10 MHz at temperatures from 25 to 200 °C. This temperature range was chosen such that a sufficiently wide temperature window is available to determine activation energies, but the upper temperature is low enough not to change the defect concentration during the electrical measurements. The complete reversibility was verified by measuring the conductivity during a complete heating up and cooling down cycle.

The DC conductance was calculated by fitting the low-frequency conduction region using Jonscher’s power law [22]:

σ= σ_DC_ + *A*ω*^n^* (1)

where σ—total conductivity, σ_DC_—the direct current conductivity of the sample, *A*ω*^n^*—pure dispersive component of AC conductivity having a characteristic of power law in terms of angular frequency ω and exponent n, and *A* is a constant that determines the strength of polarizability. All measurements were conducted in air.

## 3. Result and Discussion

**Figure 1:**
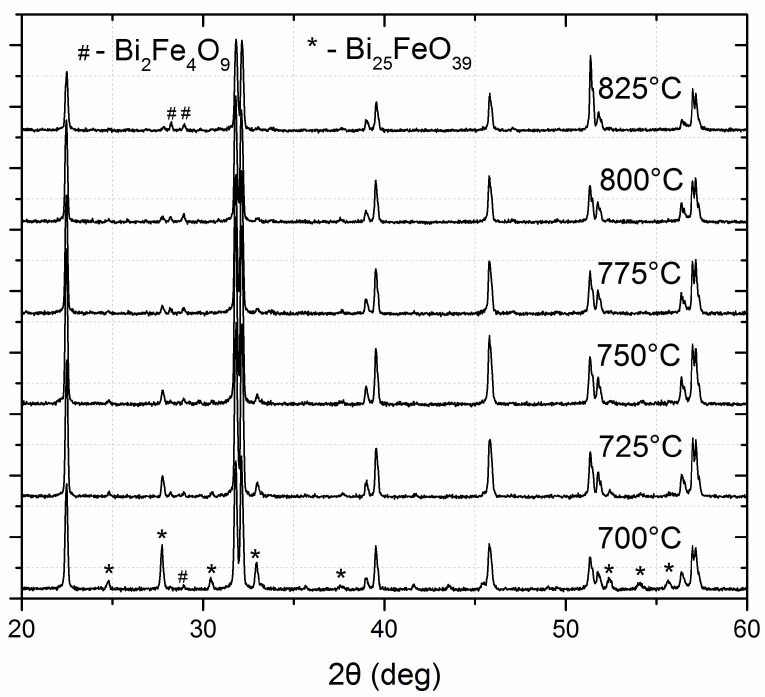
X-ray diffraction patterns of bismuth ferrite synthesized at different temperatures using stoichiometric starting material.

**Figure 2:**
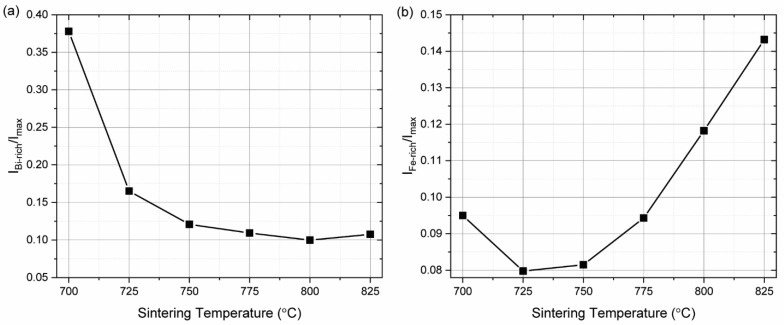
Comparison of intensities of secondary phases with the major peaks of bismuth ferrite for sample produced with stoichiometric precursors ((**a**) Bi_25_FeO_39_, (**b**) Bi_2_Fe_4_O_9_).

Figure 1 shows the X-ray diffraction patterns of BiFeO_3_ ceramics using stoichiometric starting powder calcined at different temperatures. In all samples, the major phase is the perovskite bismuth ferrite, but all samples contain substantial concentrations of the two secondary phases: one rich in bismuth—Bi_25_FeO_39_—and one iron rich—Bi_2_Fe_4_O_9_. By comparing the intensity of the most intense peaks of the secondary phases with the major peak of the bismuth ferrite (110) (Figure 2), the amount of each secondary phase present in each sample could be estimated. The peaks of Bi_25_FeO_39_ decrease with increasing synthesis temperature, while the peaks of Bi_2_Fe_4_O_9_ increase. This may be due to the loss of bismuth during the synthesis. The lowest concentration for both secondary phases was obtained for the sample calcined at 750 °C.

**Figure 3:**
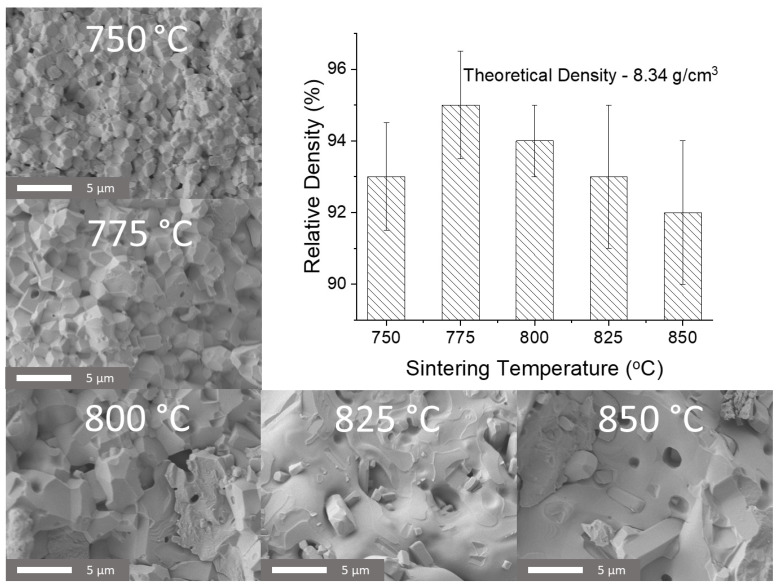
SEM micrographs of structure and density values for ceramics sintered from stoichiometric precursors at different temperatures.

Figure 3 shows the relative density and the SEM micrographs of the fracture surfaces of the samples prepared for stoichiometric precursors. For each temperature, 10 samples were prepared. SEM micrographs clearly show an increase in grain size with increasing sintering temperature. At temperatures above 825 °C, the morphology of the samples is radically different, which is due to the proximity to the melting point of Bi_25_FeO_39_ [1]. When sintering at temperatures of 825 and 850 °C, two separate microstructures can be observed to coexist: square cuboid particles and smooth shapeless crystallites. The shapeless crystallites can be attributed to bismuth ferrite and the bismuth-rich phase. The square cubic crystallites are likely to be an iron-rich phase that has a high melting point [23]. A maximum average density of 95% has been observed for the samples sintered at 775 °C.

**Figure 4:**
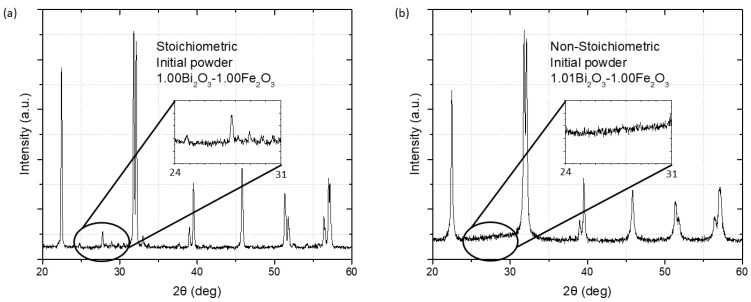
X-ray diffraction analysis of samples prepared with and without the addition of extra bismuth. ((**a**) Equal amounts of iron and bismuth in the precursor; (**b**) 1 mol% extra bismuth oxide added to precursor).

Clearly, bismuth ferrite obtained from stoichiometric precursors contains a high amount of secondary phases. Therefore, 1% excess of bismuth oxide was added to the precursors to compensate for the loss of Bi, which might occur during the sintering process to increase the phase purity of bismuth ferrite. The XRD pattern of the sample calcined and sintered from the stoichiometric precursors (Figure 4a) shows both secondary phases, Bi_25_FeO_39_ and Bi_2_Fe_4_O_9_. However, in the XRD pattern of the sample with excess bismuth (Figure 4b), these secondary phases and their diffraction peaks are not detectable at the level of background noise.

**Figure 5:**
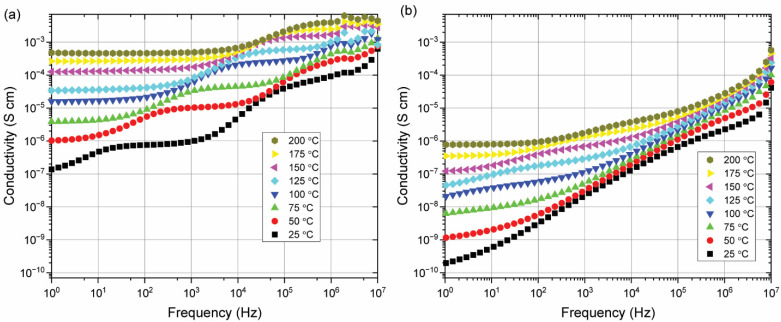
AC Conductivity measurements ((**a**) From stoichiometric precursor BiFeO_3_, (**b**) from 1% Bi_2_O_3_ enriched precursor BiFeO_3_).

Figure 5 shows the results of impedance data for different temperatures for the stoichiometric and Bi-enriched samples that correspond to the XRD patterns shown in Figure 4. From these spectra, two processes that contribute to the electrical conductivity are noticeable. While the nature of these processes is still under discussion, there is a noticeable decrease in the conductivity of the phase pure sample when compared to the sample that contains secondary phases. This may be associated with a decrease in the concentration of bismuth and oxygen vacancies with the addition of extra bismuth or the absence of an easy conductivity path via the secondary phases.

**Figure 6:**
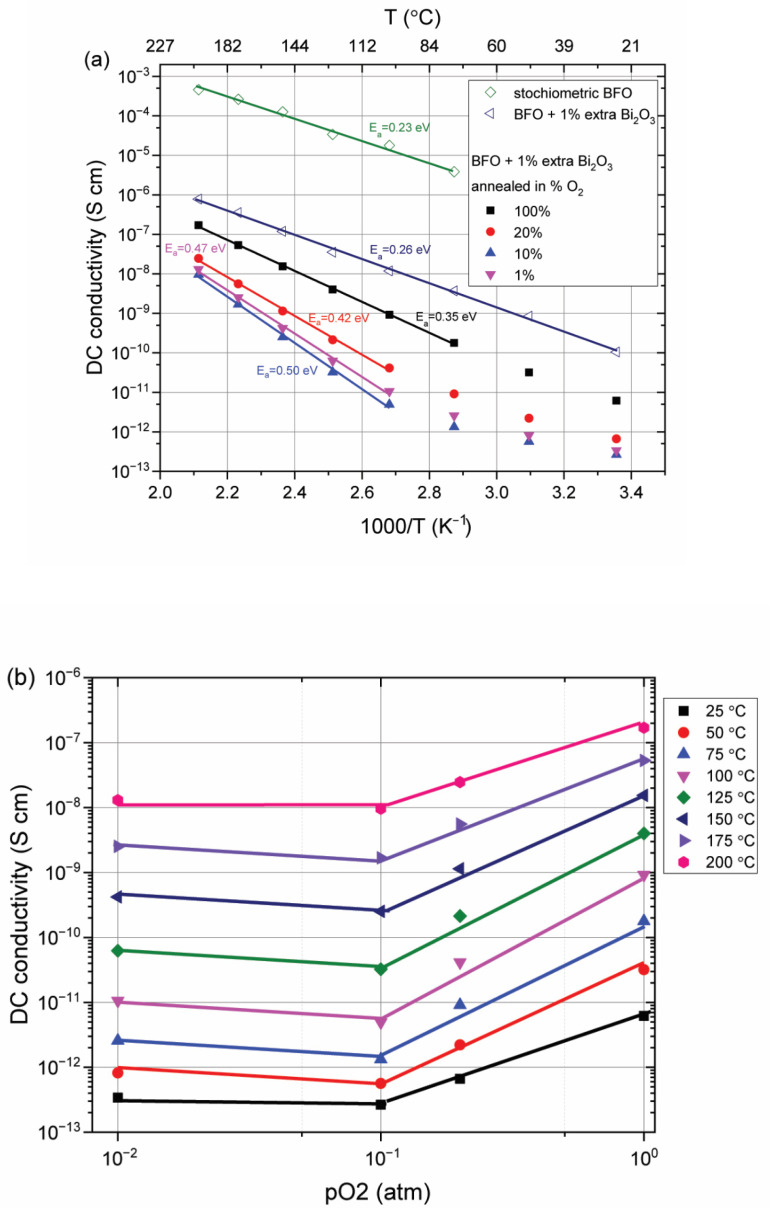
Conductivity of BFO samples prepared and annealed in different atmospheres. (**a**) Arrhenius plot of DC conductivity as a function of inverse temperature for reheated samples after annealing and linear cooling from 750 °C. (**b**) DC conductivity measured in air over the temperature range from 25 to 200 °C for samples synthesized from non-stoichiometric precursors for various oxygen partial pressures during high-temperature processing.

In Figure 6, the Arrhenius plot is given for the DC conductivity using samples prepared from the stoichiometric and non-stoichiometric precursors. The DC conductivity in the pure phase sample synthesized from the non-stoichiometric precursor is significantly lower than the conductivity of the samples containing secondary phases. It is clearly visible from Figure 6 that for both materials, the conductivity is a thermally activated process with a similar activation energy of about 0.25 eV. This strongly suggests that the conductivity mechanism for both samples is of the same nature.

Investigation of the effect of defect concentration on electrical conductivity was performed by annealing pure BiFeO_3_ ceramic samples in gasses with different oxygen content. As shown in Figure 6a, a decrease in conductivity with decreasing oxygen concentration in the gas environment during annealing can be observed, which is not surprising for a material demonstrating p-type conductivity. It should be noted that the conductivity decreases very rapidly with a relatively small decrease in oxygen in the gas mixture. The activation energy of that conduction mechanism increases with a decrease in oxygen pressure leading to much higher values than for the as-sintered samples.

The oxygen vacancies involved in the conduction mechanism may be associated with the volatility of Bi_2_O_3_:

(2) $$2Bi_{Bi}^{X}+3O_{O}^{X}\to 2V{{}^{\prime \prime \prime }}_{Bi}+3V_{O}^{\cdot \cdot }+{\mathrm{Bi}}_{2}O_{3}$$

where $Bi_{Bi}^{X}$—Bi position occupied by Bi, $O_{O}^{X}$—O position occupied by O, $V{{}^{\prime \prime \prime }}_{Bi}$—bismuth vacancy, $V_{O}^{\cdot \cdot }$—oxygen vacancy. The hole concentration was controlled by annealing and can be described by the following reaction:

(3) $$2V_{O}^{\cdot \cdot }+O_{2}\to 2O_{O}^{X}+4h^{\cdot }$$

where $h^{\cdot }$—electron hole and $e^{\prime }$—free electron. As the oxygen concentration in the atmosphere decreases, the balance shifts to the left, reducing the concentration of free charge carriers, and as a result, the overall conductivity. This behavior is confirmed in the experiments, confirming the p-type conductivity in the material. Hole conduction behavior in BiFeO_3_-based ceramics was previously attributed to oxidation of Fe^3+^ to Fe^4+^ [24]. However, excess Bi_2_O_3_ was introduced in the starting materials of BFO to suppress its volatility during processing. The Bi non-stoichiometry can further lead to a significant change in conduction behavior. As a result, the oxygen loss can be compensated by electrons:

(4) $$2O_{O}^{X}\to 2V_{O}^{\cdot \cdot }+O_{2}+4e^{\prime }$$

leading to an n-type conductivity, which is not observed here.

As shown in Figure 6a, samples that were annealed at different partial oxygen pressures show two trends in conductivity (from RT till around 100 °C and from 100 °C to 200 °C). This can be explained by the fact that the concentration of carriers generated by impurities is much higher than that of thermally generated intrinsic carriers at temperatures below 100 °C, and the effect of mobility reduction caused by electron–phonon collisions is small. The concentration of charge carriers generated from impurities weakly depends on temperature, and the slope can be explained by the change in their mobility during heating; therefore, the electronic conductivity decreases slightly when cooled due to the low mobility of these charges. Samples not having received this post-synthesis annealing treatment do not show this dual conduction behavior, most likely due to the very high concentration of charge carrier impurities. The activation energy of conduction increases for annealed samples due to a decrease in the concentration defects or compensation of free charge carriers.

Figure 6b shows the pO_2_ dependence of DC conductivity for the pure phase BFO. As can be seen, for the pO_2_ region greater than 0.1 atm, the total conductivity exhibits a positive slope when plotted against the oxygen concentration, which reflects the hole contribution. For low oxygen concentrations, the conductivity becomes independent of the oxygen concentration, which indicates an increased contribution of ion conduction [24].

**Figure 7:**
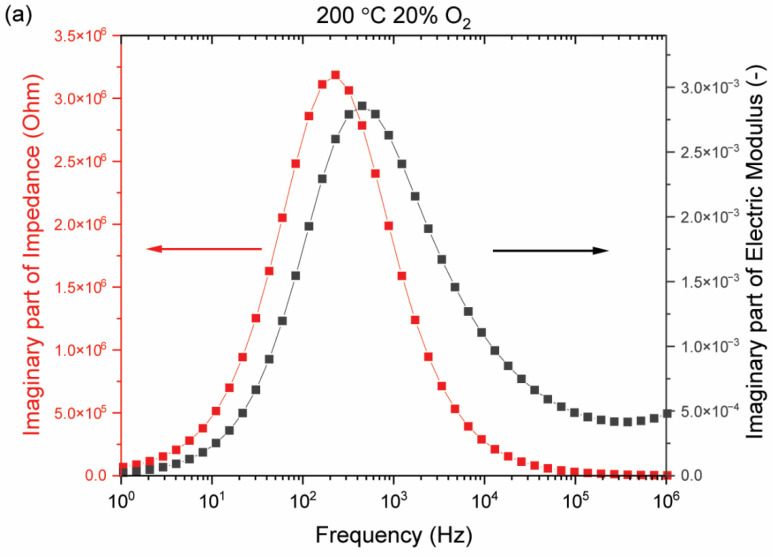

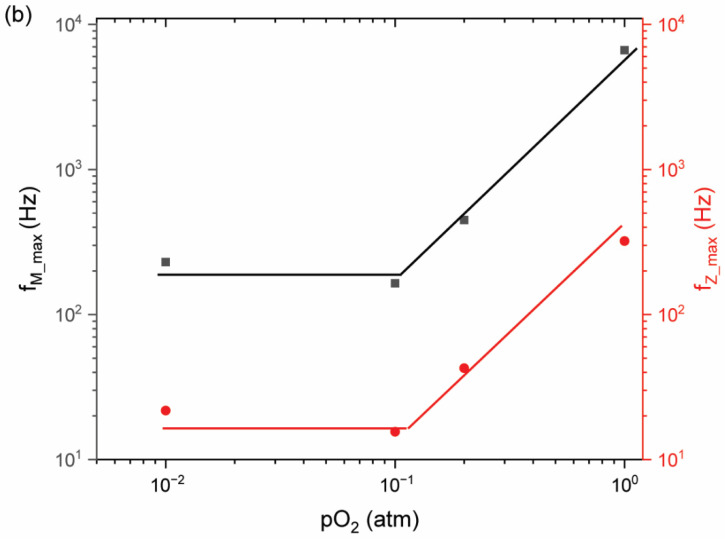
Results of impedance spectroscopy analysis for data measured at 200 °C. (**a**) Impedance and electric modulus spectroscopic plots for samples annealed in 20% O_2_; (**b**) electrical modulus peak and impedance peak frequency as a function of pO_2_.

A comparison of the impedance with the electric modulus data allows the determination of the bulk response in terms of localized or non-localized conduction [16]. In Figure 7a, the imaginary part of the electrical modulus and the imaginary part of impedance as a function of frequency at 200 °C are shown. The overlap of the peak positions of the M” and Z” curves is evidence of delocalized or long-range relaxation [16]. However, for the present, the peaks do not overlap perfectly but are very close, suggesting that conduction contains components from both long-range and localized relaxations. Figure 7b shows the electrical modulus and impedance peak frequency as a function of pO_2_ at a fixed measurement temperature of 200 °C. As can be seen, the frequency shift for the electrical modulus and impedance does not change with a change in the oxygen concentration, which can serve as an indirect confirmation of the assumption that oxygen-related defects dominate the conductivity of the obtained samples and that the contribution of other conductivity mechanisms is small. In other studies, this is presented as evidence of a long-range conductivity range for free oxygen vacancies [25,26]. The dependence of the maximum frequency of the peaks of the electric modulus and impedance on pO_2_ is in complete agreement with the inferred dependence of the conductivity on defects and the actual lattice.

As stated earlier, measurements were made at temperatures below 200 °C to ensure that the defect population remains the same and only reflects the state resulting from the annealing condition (annealing at a temperature of 750 °C followed by linear cooling under controlled oxygen partial pressure). Given that thermal cycling in the air up to a temperature of 200 °C does not change the conductivity, we indirectly established that the defect state in the samples was established at a temperature between 750 °C and 200 °C. Our experiments were not set up to properly establish the exact conditions at which the defect state reached its ‘frozen’ state, nor could it be established whether the ‘frozen’ state was determined by thermodynamics or kinetics. The frozen state obtained at a partial oxygen pressure below 0.1 atm shows a purely ionic nature, which shows us that the generation of oxygen vacancy defects has reached saturation point with the concentration of charge carriers remaining the same. This fully confirms our assumption that the nature of the high conductivity is associated with oxygen vacancies.

## 4. Conclusions

Pure BiFeO_3_ with a relatively low electrical conductivity can be obtained by conventional solid-state sintering at 750 °C starting from a non-stoichiometric composition of precursors. Adding 1% excess of Bi_2_O_3_ compensates for the loss of bismuth during synthesis and sintering. The absence of secondary phases resulted in a reduction of the electrical conductivity by more than a factor of 1000. Annealing pure BiFeO_3_ in mixtures of nitrogen and oxygen gases at various oxygen partial pressures reduced the conductivity further. Hence, control of the oxygen partial pressure in the atmosphere during high-temperature processing is a novel tool to manipulate the oxygen-related defects, which play a major role in the conductivity of BiFeO_3_. In case the oxygen concentration in the gaseous environment is below 10%, the conductivity seems to be determined by other intrinsic charge carriers.

## Figures and Tables

**Table 1:** Flows rates of oxygen and nitrogen for the different annealing.

O_2_ (%)	N_2_ Flow (mL/min)	O_2_ Flow (mL/min)	Total Flow (mL/min)
1	2970	30	3000
10	225	25	250
20	200	50	250
100	0	250	250

## Supplementary Materials

The following supporting information can be downloaded at: www.mdpi.com/article/10.3390/ma15196509/s1, Figure S1: Photo of the sample after electrical measurement with gold electrodes, Figure S2: Block diagram of the synthesis and sintering of BiFeO3 and its subsequent analysis to optimize the preparation process, Figure S3: Block diagram of the synthesis and sintering of bismuth ferrite and subsequent annealing, to study the effect of oxygen defects on conductivity.
