# Supplementary material for: Controlling the Oxygen Defects Concentration in a Pure BiFeO3 Bulk Ceramic
Source: Materials (Basel). 2022 Sep 20;15(19):6509. doi: 10.3390/ma15196509 (PMC9571174; doi:10.3390/ma15196509)
Supplement: Supplementary file 1 [file materials-15-06509-s001.zip › materials-1917415-supplementary.pdf]

Supplementary materials

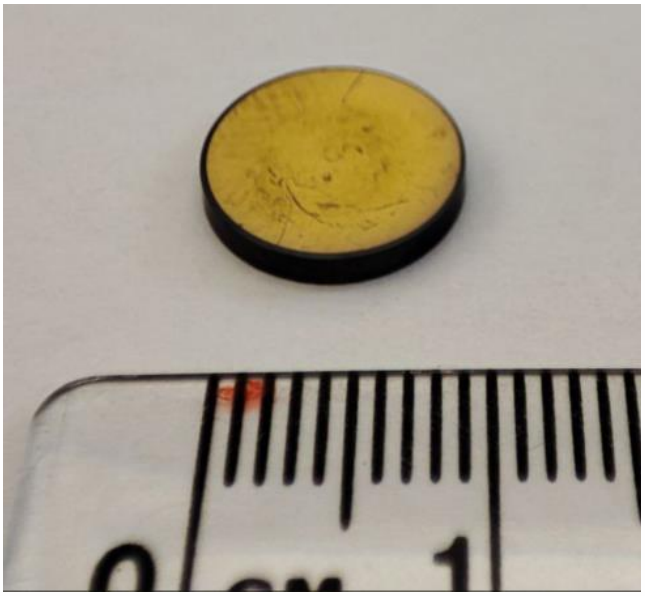

Figure S1 – Photo of the sample after electrical measurement with gold electrodes

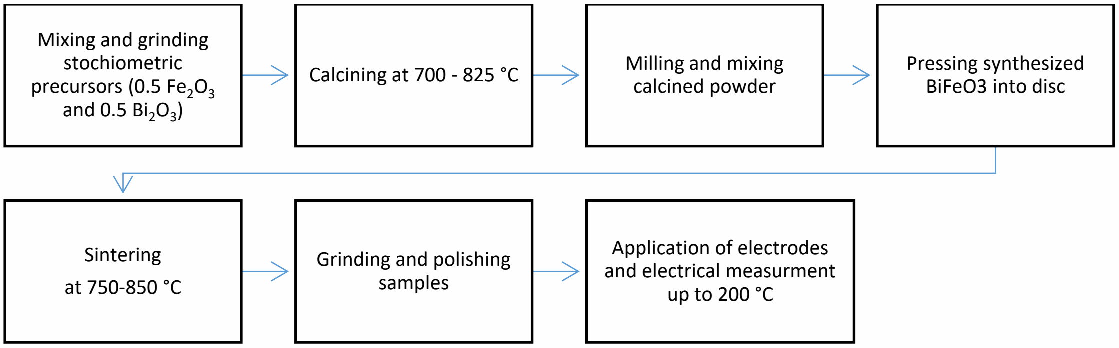

Figure S2 – Block diagram of the synthesis and sintering of $BiFeO_3$ and its subsequent analysis to optimize the preparation process

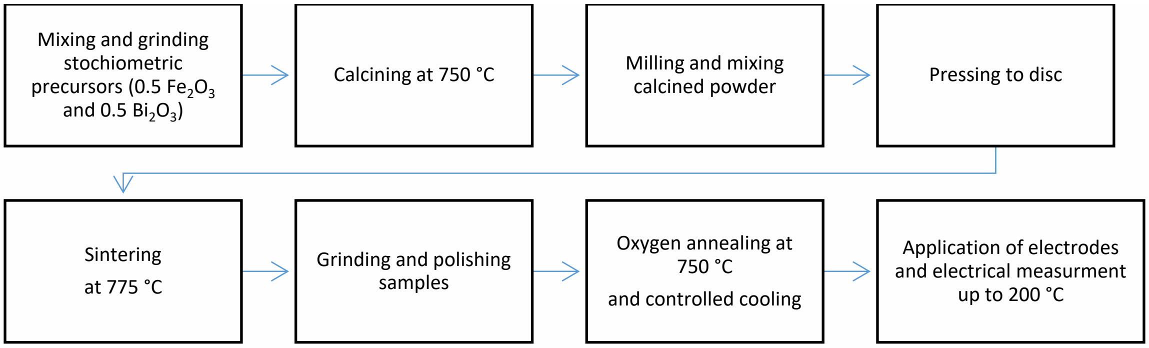

Figure S3 – Block diagram of the synthesis and sintering of bismuth ferrite and subsequent annealing, to study the effect of oxygen defects on conductivity
